# Endurance of larch forest ecosystems in eastern Siberia under warming trends

**DOI:** 10.1002/ece3.2285

**Published:** 2016-07-22

**Authors:** Hisashi Sato, Hideki Kobayashi, Go Iwahana, Takeshi Ohta

**Affiliations:** ^1^ Department of Environmental Geochemical Cycle ResearchJapan Agency for Marine‐Earth Science and Technology (JAMSTEC)3173‐25 ShowamachiKanazawa‐kuYokohama236‐0001Japan; ^2^ International Arctic Research CenterUniversity of Alaska FairbanksPO Box 757340930 Koyukuk DriveFairbanksAlaska99775; ^3^ Graduate School of Bioagricultural SciencesNagoya UniversityFuro‐choChikusa‐kuNagoya464‐8601Japan

**Keywords:** Climate change, land surface model, larch forests, permafrost, SEIB‐DGVM, thermo‐hydrology

## Abstract

The larch (*Larix* spp.) forest in eastern Siberia is the world's largest coniferous forest. Its persistence is considered to depend on near‐surface permafrost, and thus, forecast warming over the 21st century and consequent degradation of near‐surface permafrost is expected to affect the larch forest in Siberia. However, predictions of these effects vary greatly, and many uncertainties remain about land – atmosphere interactions within the ecosystem. We developed an integrated land surface model to analyze how the Siberian larch forest will react to current warming trends. This model analyzed interactions between vegetation dynamics and thermo‐hydrology, although it does not consider many processes those are considered to affect productivity response to a changing climate (e.g., nitrogen limitation, waterlogged soil, heat stress, and change in species composition). The model showed that, under climatic conditions predicted under gradual and rapid warming, the annual net primary production of larch increased about 2 and 3 times, respectively, by the end of the 21st century compared with that in the previous century. Soil water content during the larch‐growing season showed no obvious trend, even when surface permafrost was allowed to decay and result in subsurface runoff. A sensitivity test showed that the forecast temperature and precipitation trends extended larch leafing days and reduced water shortages during the growing season, thereby increasing productivity. The integrated model also satisfactorily reconstructed latitudinal gradients in permafrost presence, soil moisture, tree leaf area index, and biomass over the entire larch‐dominated area in eastern Siberia. Projected changes to ecosystem hydrology and larch productivity at this geographical scale were consistent with those from site‐level simulation. This study reduces the uncertainty surrounding the impact of current climate trends on this globally important carbon reservoir, and it demonstrates the need to consider complex ecological processes to make accurate predictions.

## Introduction

**Figure 1:**
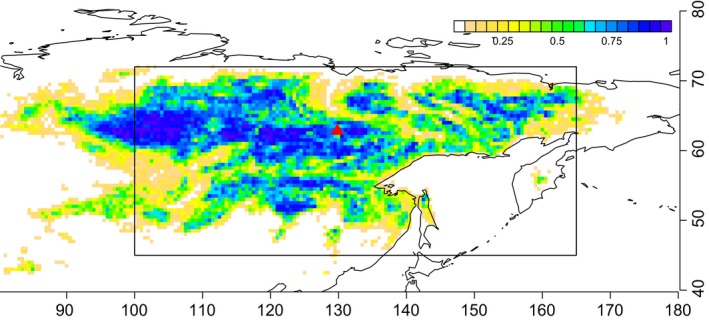
The geographical distribution of larch forest in eastern Siberia. The color contours indicate the fraction of larch forest in each 0.5° grid square, taken from the Global Land Cover 2000 data sets (IES‐Global‐Environment‐Monitoring‐Unit 2003). The red triangle shows the location of the study site at Sppaskaya‐pad. The square box indicates the area used for the latitudinal gradient analysis. The vertical axis shows latitude and the horizontal axis longitude.

In eastern Siberia, larches (*Larix* spp.) often exist in pure stands that are important components of the world's largest coniferous forest (Fig. 1), which is regenerated by recurrent fire (Schulze et al. 1995). This region is characterized by a short, dry growing season: the mean annual precipitation in meteorological observatory in Yakutsk is around 240 mm, and evapotranspiration efficiency (fraction of actual evapotranspiration rate to its potential maximum) is 0.30–0.45 (Ohta et al. 2008). Thus, the underlying permafrost and seasonal freeze–thaw cycle, which inhibit percolation of water into deep soil layers, are considered to be important in maintaining water flow through forest ecosystems in this arid environment (Horiguchi and Miller 1980; Jorgenson et al. 2010). Moreover, the geographical distribution of larch forests in southeast Siberia closely matches the permafrost zone, supporting the suggestion that the forest depends on permafrost (Abaimov 2010).

Over the 21st century, high‐latitude regions are predicted to experience intense warming (IPCC, 2013), which may cause near‐surface permafrost to disappear across Siberia (Lawrence et al. 2012). Moreover, a bioclimatic study of permafrost and fire activity showed that the forecast warming could cause larch forest systems to shift to forest–steppe or steppe systems in southeast Siberia (Tchebakova et al. 2009). However, to our knowledge, only two previous investigations projecting the resilience of larch forests under warming trends in Siberia (Beer et al. 2007 and Zhang et al. 2011) have taken a process‐based approach that directly considers interactions between vegetation dynamics and permafrost.

The first of these studies adapted the Lund–Potsdam–Jena Dynamic Global Vegetation Model (DGVM) for Siberia by incorporating a freeze–thaw process and found that vegetation carbon density was overestimated by 2–5 times when freeze–thaw processes were not considered (Beer et al. 2007). One major reason for this overestimation was water availability. In the initial model, runoff only occurred when soil water content exceeded field capacity in all unfrozen soil layers. This assumes that water is retained as if it were in a bucket and is only lost when it exceeds a maximum volume, which decreases with the number of frozen soil layers. Thus, overlooking freeze–thaw processes artificially increased the soil's water storage capacity.

Conversely, in the vegetation model used by Zhang et al. (2011), soil water drains rapidly when near‐surface permafrost thaws, causing a large water deficit in summer and consequent collapse of the Siberian larch forest. Their model described the vertical profile of soil moisture through a more complex calculation that considered the matrix potential of soil and vertical vapor diffusion. In a sensitivity experiment, this model showed that larch forests in eastern Siberia did not persist when temperature increased by only approximately 2°C (Zhang et al. 2011). The process‐based models of Beer et al. (2007) and Zhang et al. (2011) thus differ markedly in what influence decaying near‐surface permafrost is expected to have on ecosystems, leaving significant uncertainty about how the Siberian larch forest will change under current warming trends.

To address this uncertainty, we have here further developed the process‐based approach to modeling the response of Siberian larch forest to warming by integrating the Spatially‐Explicit Individual‐Based DGVM (SEIB‐DGVM; Sato et al. 2007) with the Noah Land Surface Model (NOAH‐LSM; Ek et al. 2003), which incorporates freeze–thaw processes. This integrated model allows consideration of a key difficulty in simulating how permafrost affects vegetation, namely that the vegetation also affects surface permafrost in numerous ways. Vegetation can alter permafrost dynamics through processes including canopy shading, snow interception, and litter accumulation, which inhibit heat exchange between the soil and atmosphere (Jorgenson et al. 2010). For example, in permafrost ecosystems in Alaska, canopy removal following fire increased both ground heat flux (Chambers and Chapin 2003; Liu et al. 2005) and active layer depth (ALD) (Viereck 1982). Accordingly, our integrated model considered two dominant influences of vegetation on freeze–thaw processes: (1) interference in soil–atmosphere heat exchange by a litter layer and (2) attenuation of sunlight by foliage. Previous studies have considered either the former process (Beer et al. 2007) or the latter (Zhang et al. 2011) but not both.

To validate our new integrated model, we examined whether it reconstructed observed patterns in key aspects of vegetation dynamics and thermo‐hydrology. We then employed the validated integrated model to (1) predict whether Siberian larch forests can establish and develop under forecast climatic conditions during the 21st century, (2) identify which combination of climatic variables has the greatest influence on simulated changes using sensitivity tests, and (3) evaluating how general model reliability is using simulations across a geographic area containing most of the larch‐dominated region in eastern Siberia.

## Methods

### Model

**Figure 2:**
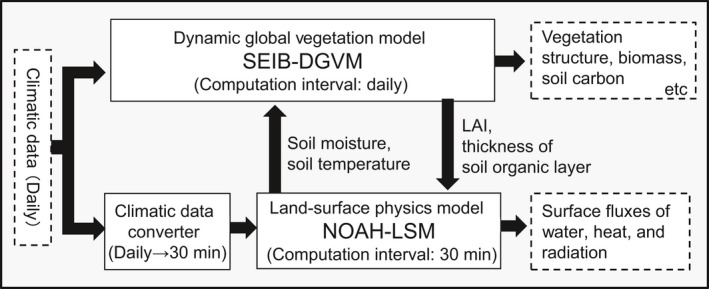
A schematic diagram of the integrated model developed for this study. Solid boxes show submodels, and dashed boxes and labeled arrows represent climate variables and ecosystem functions.

The integrated model we developed for this study is shown schematically in Figure 2. One of the two model components is the SEIB‐DGVM (Sato et al. 2007), which simulates plant and carbon dynamics under specified climatic conditions (further details in Appendix S1). Because SEIB‐DGVM does not consider surface heat balance, it cannot infer soil freeze–thaw cycles, which are the main regulator of seasonal changes in soil water availability for plants in the study region. To account for this, a previous simulation of the larch forest in Yakutsk by Sato et al. (2010) used SEIB‐DGVM to force a simulated meteorological data set with a land surface physical model (Yamazaki et al. 2007). In this study, we introduced the one‐dimensional (i.e., not considering the lateral flow of water and heat) land surface model NOAH‐LSM 2.7.1 (Ek et al. 2003) to mechanistically link freeze–thaw cycles with forest succession. This model simulated soil moisture (both liquid and frozen), soil temperature, snowpack depth and density, canopy water content, and energy and water fluxes under specified climatic and soil conditions. The ability of NOAH‐LSM to reproduce hydrological and thermal processes in regions with permafrost has been validated in several studies (e.g., Van Der Velde et al. 2009; Yang et al. 2009; Chen et al. 2011; Zeng et al. 2012), such that using this model was preferable to developing an original thermo‐hydrological submodel, as was performed by Beer et al. (2007) and Zhang et al. (2011).

In this integrated model, exchange variables in SEIB‐DGVM and NOAH‐LSM were updated daily. NOAH‐LSM provided SEIB‐DGVM with daily averages of unfrozen soil moisture content and temperature for each soil layer, and SEIB‐DGVM provided NOAH‐LSM with three parameters: (1) thermal conductivity of the top soil layer (*df*_1_) as a function of litter mass on the forest floor; (2) green vegetation fraction (GVF), defined as GVF = log(4.5 LAI + 1), where LAI is the leaf area index (leaf area per ground area; m^2^ m^−2^); and (3) LAI of the green vegetation area (*xlai*), which is calculated as LAI divided by the GVF. The green vegetation fraction is assumed to reach 1.0 when LAI reaches 2.0 in the previous equation. Further details concerning model integration and settings are given in the Appendix S1.

### Simulation location

Intensive simulations in this study were based on a larch forest at the Spasskaya‐pad Scientific Forest Station (Fig. 1; 62.8150°N, 129.8370°E; 220 m above sea level), where meteorological and hydrological data have been collected since 1998 (Ohta et al. 2008). This region is dominated by Siberian larch, *Larix gmelinii*, with a mean stand height of 18 m, stand density of 808 trees ha^−1^, and age of 160 years (Iijima et al. 2014). The forest floor vegetation is composed predominantly of birch (*Betula platyphylla*), willow (*Salix bebbiana*), and evergreen shrubs (*Vaccinium vitis‐idaea* and *Arctostaphylos uva‐ursi*) (Suzuki et al. 2001; Shibuya et al. 2004).

### Climate data

The following climate data were used to force the model: daily mean air temperature, air temperature range, precipitation, downward shortwave and longwave radiation, nondimensional wind velocity, and specific humidity (Table S2; predicted trajectories in Fig. S1).

To calibrate and validate the model for the Spasskaya‐pad site, we repeatedly input 41 years (1966–2006) of climate data, using a constant atmospheric CO_2_ concentration of 368 ppm (the value observed in 2000). These climate data were generated from observations at Yakutsk city (62.08°N, 129.75°E; 101 m above sea level) by Baseline Meteorological Data in Siberia Version 5 (BMDS5; Yabuki et al. 2011) (details in Appendix S1).

We then projected the behavior of Siberian larch forests in Spasskaya‐pad over the 21st century and conducted simulations of the entire larch‐dominated area under both current and future climatic conditions. This modeling used output from the Model for Interdisciplinary Research on Climate Earth System Model (MIROC‐ESM; Watanabe et al. 2011) based on the Intergovernmental Panel on Climate Change (IPCC) Representative Concentration Pathway (RCP) for greenhouse gas (GHG) concentrations, using RCP scenarios 8.5 and 2.6. To ensure consistency between data sets, MIROC data were linearly scaled by multiplying each possible combination of month, climatic parameter, and RCP by a constant (or adding a constant, in the case of air temperature) so that the monthly averages from 1966 to 2006 equaled the corresponding monthly averages of the observed data over the same period. In addition, we expanded the monthly MIROC data with diurnal variability derived from the observational climate data. To do this, daily deviations were applied to the monthly data that were derived by dividing (or subtracting in the case of air temperature) all daily climatic factors by the average of corresponding months from 1966 to 2006.

### Model validation

After calibrating the model (see Appendix S1 for details), we examined whether it successfully reconstructed the following key aspects of thermo‐hydrology and vegetation dynamics at Sppaskaya‐pad: (1) postfire vegetation dynamics, (2) seasonal changes in ecosystem functions in a mature forest, (3) annual variability in hydrological characteristics in mature forests, and (4) changes in soil thermo‐hydrological status after clear‐cutting. Here, we only present outcomes for aspects 3 and 4 (items 1 and 2 are addressed in the Appendix S1).

Hydrological characteristics are important because soil water content has been shown to be the primary determinant of interannual variability in evapotranspiration efficiency during the growing season in the same biotope (Ohta et al. 2008). In this forest, water availability has been shown to limit larch productivity, with soil water deficits during the growing season inhibiting vegetation productivity by 50% (Dolman et al. 2004).

We calculated potential evapotranspiration rate using methods proposed by Kondo and Xu (1997) and Xu and Haginoya (2001). To force this simulation, we input 41 years (1966–2006) of climate data four times and examined results for 1998–2006, where tower‐observed flux data are available. Thus, the simulation treated the forest as being 155 years old, whereas the observed forest was estimated to be 160 years old (Dolman et al. 2004). Fire was not allowed to occur during the simulation.

We also validated the model against observed changes in soil water content after clear‐cutting. Although both clear‐cutting and stand‐replacing fire remove all trees, the former does not remove the organic layer on the forest floor (Iwahana et al. 2005), which works as an efficient heat insulator between the soil and atmosphere (Saito et al. 2004; Yi et al. 2007; Lawrence and Slater 2008; Johnson et al. 2013). Therefore, clear‐cutting should affect the thermo‐hydrological cycles only via changes in radiation balance and evapotranspiration efficiency, with heat exchange conductance of the forest surface remaining the same as that of the mature forest.

The influence of clear‐cutting was examined using a larch forest site near Yakutsk, where all the trees in a rectangular site (70 × 170 m) were removed at the end of 2000 to assess the impact of clear‐cutting on soil thermo‐hydrological cycles (Iwahana et al. 2005). Soil thermo‐hydrological measurements were compared between the clear‐cut site and an intact forest plot located about 100 m away. To reproduce this field experiment with our model, we conducted a clear‐cut run and compared it with a control run. In the clear‐cut run, all trees were removed on December 1, 2000, and the soil organic layer remained intact. In the clear‐cut run, NOAH‐LSM parameters were also altered after the clear‐cutting (Table S1). Both the clear‐cut and control runs were forced using 41 years (1966–2006) of observation‐based climate data, input five times, and the results of the last repeat in 2001 and 2002, where observed data are available, were examined. Thus, the simulations assumed 199‐year‐old forests, whereas the forests at our study site were estimated to be over 200 years old.

### Simulations under forecast climatic conditions

We used the validated model to examine whether larch forests in eastern Siberia can establish and develop under the climatic conditions predicted for the 21st century by the MIROC atmospheric general circulation model (AGCM) (K‐1‐Model‐Developers, 2004; Emori et al. 2005) with IPCC RCP scenarios 8.5 and 2.6 for rising CO_2_. The status of larch forest was examined for each base climatic year from 1951 to 2091. Each year was simulated 10 times using 10 years of climatic data, starting from the base year (i.e., 100 years of simulation), and averages of the last 10 years of the simulation were analyzed. In addition, we conducted another 100‐year simulation as a spin‐up phase by inputting sequential climate data from the last 100 years. We used MIROC‐AGCM historical climate data until 2005 and projections from MIROC‐AGCM for 2006–2100.

### Sensitivity to the changing environment

To determine the main climatic causes of the simulated changes to larch forests, we performed a sensitivity test and evaluated the effect of each environmental variable separately. For this analysis, we conducted a 251‐year simulation from 1850 to 2100, which consisted of a 151‐year spin‐up phase (1850–2000) and a 100‐year build‐up phase (2001–2100). A stand‐replacing fire was included between the phases (i.e., at the end of 2000), whereas no fire was allowed to occur during either phase. For the build‐up phase, we repeatedly input data from 1991 to 2000 for all environmental variables except the variable that was being evaluated. For these target variables, we input projections from 2001 to 2100 under RCP 8.5 conditions. For the analysis, we compared the averages of the last 10 years of the spin‐up phase (1991–2000) and the build‐up phase (2091–2100). Air temperature and precipitation caused marked changes to the result, and we therefore examined their interactive influence by manipulating them simultaneously.

### Simulations for the entire larch‐dominated area

To evaluate the generality of the model outputs, we conducted simulations across a geographic area that contains most of the larch‐dominated region in eastern Siberia (45–72°N and 100–165°E; Fig. 1). This larch‐dominated region is defined by the frequency in each 0.5° grid square of the “deciduous needleleaf forest” category in the Global Land Cover 2000 data sets (IES Global Environment Monitoring Unit 2003). As our model only includes Siberian larch species for tree plant functional type (PFT), we excluded the western border of the larch‐dominated region, which is a zone of transition to evergreen coniferous forest. This simulation used Zobler's global soil types (Zobler 1999), a gridded global data set of soil types at a resolution of 1°, but other parameters were kept the same as for the Sppaskaya‐pad simulations.

For each grid cell within the simulation range, a 1000‐year spin‐up run was first conducted. Climate data for 1850 to 1879 were repeatedly input and atmospheric CO_2_ concentration was assumed to be 285 ppm, the global mean value in 1850. After the spin‐up phase, we conducted simulations for 1851–2100 (250 years) with continuous climatic data generated from the MIROC‐ESM under RCP 8.5. Atmospheric CO_2_ concentrations were global historical data until 2005 and RCP 8.5 projections from 2006.

We also evaluated how soil‐freezing process and surface permafrost influence hydrology and hence plant productivity in “NoIce” and “NoIce‐NoLeak” runs. In the “NoIce” run, soil water was not allowed to freeze, while in the “NoIce‐NoLeak” run, neither soil water freezing nor bottom flow were allowed.

## Results

### Model validation under current climatic conditions

**Figure 3:**
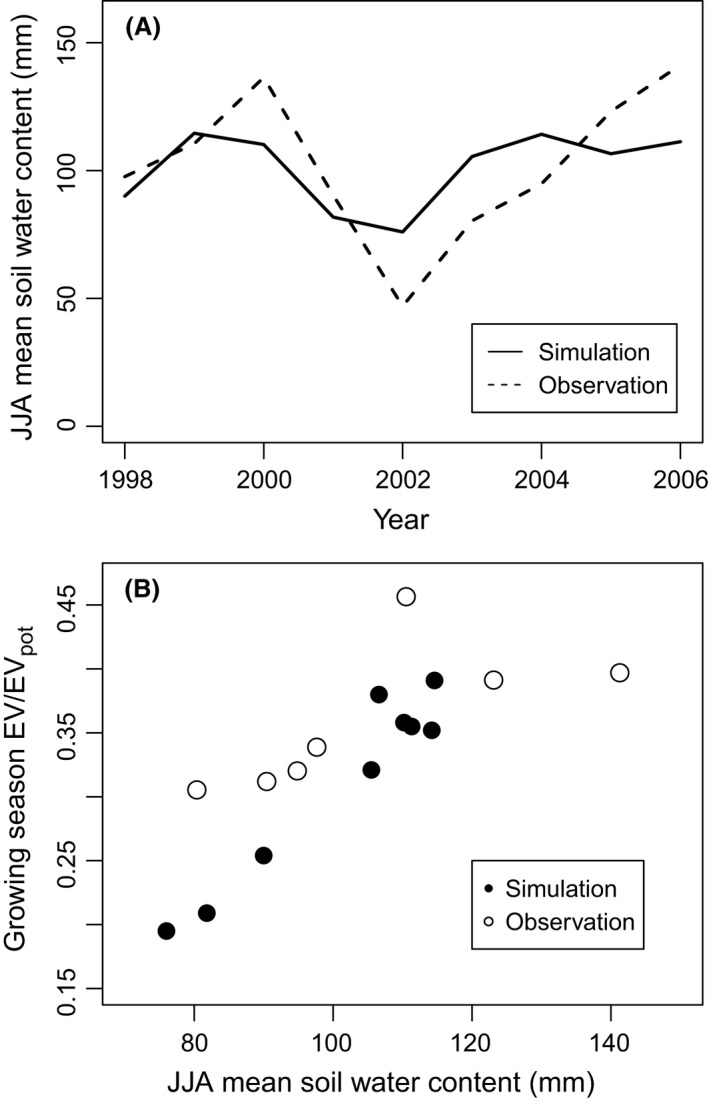
Examples of model validation. All values are averages from June, July, and August (JJA), which is the larch‐growing season at the study site. Observed values were taken from Ohta et al. (2008). (A) Comparison of simulated and observed interannual variation in soil water content of the 0‐ to 50‐cm soil layer. (B) Interannual variation in evapotranspiration efficiency (actual evapotranspiration rate divided by its potential maximum).

After calibration, the model successfully reconstructed interannual variability in soil hydrological characteristics (Fig. 3A) and the consequent evapotranspiration efficiency (Fig. 3B) of a mature larch forest under current climatic conditions. Reconstructions of interannual variability should not be directly delivered through our calibration process, as the calibration was only conducted for the model to reconstruct postfire developments of thermal conductivity in the top soil layers (0–10 cm) and forest structure in terms of tree density and aboveground biomass (AGB).

In the field experiment, clear‐cutting increased soil water content considerably by reducing transpiration rate during the growing season. Observed average soil water content (0–50 cm depth) from June to August in 2001 and 2002 was 20.7% and 27.7% in intact and clear‐cut forest plots, respectively (Iwahana et al. 2005). The simulation gave corresponding values of 15.5% and 21.4%, such that clear‐cutting increased soil water content by 7.0% in the field experiment and by 5.9% in the simulation. Although baseline soil water differed by about 5% between the field experiment and simulation, the outcome of clear‐cutting was comparable.

The clear‐cut manipulation also increased ALD by eliminating the canopy's interception of solar radiation. The average maximum thaw depths in 2001 and 2002 were 1.04 m and 1.19 m for intact and clear‐cut forest plots, respectively (Iwahana et al. 2005), with corresponding simulated values of 0.76 m and 1.38 m. Our integrated model thus apparently overestimated increases in ALD after clear‐cutting.

The model also reconstructed the postfire development of net primary production (NPP; g dry mass tree^−1^ year^−1^) of larch trees and seasonal changes in NPP and net ecosystem exchange (NEE) in mature forests reasonably well (Appendix S1).

### Simulations under forecast climatic conditions

**Figure 4:**
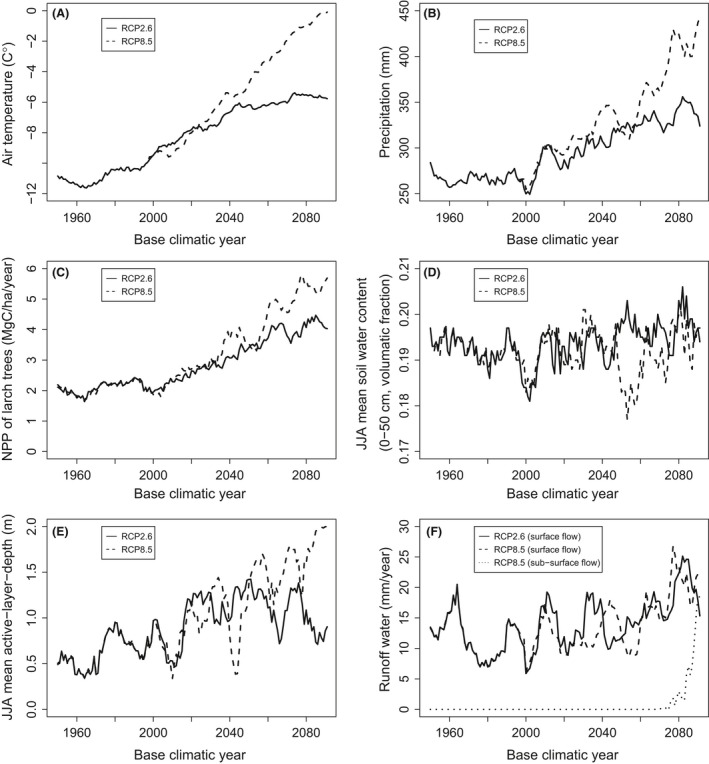
Forecast climatic conditions (A,B) and their influence on the simulated larch forest (C–F) at Sppaskaya‐pad. Climatic conditions show running means of the last 10 years of the base climatic year. For simulation results, a 200‐year simulation was conducted, and averages of the last 10 years of the results are presented. The simulation consisted of a 100‐year spin‐up phase followed by a 100‐year build***‐***up phase. Stand‐replacing fire occurred between the spin**‐**up and build‐up phases, but fire was not allowed to occur during each phase. The panels show (A) annual mean air temperature; (B) annual precipitation; (C) annual NPP of larch trees; (D) average soil water content within the 0‐to 50‐cm soil layer from June to August; (E) average ALD from June to August; and (F) runoff (surface or subsurface flow). Subsurface runoff only occurred under RCP 8.5, and thus, only surface runoff was presented for RCP 2.6.

Under the climatic conditions forecast by MIROC for the 21st century, our integrated model shows a trend of increasing larch productivity (Fig. 4). Simulated annual larch NPP under RCP 2.6 and 8.5 are, respectively, about 2 and 3 times higher under the climatic conditions at the end of 21st century than of the 20th century. In contrast, June to August soil water contents showed no obvious trend (Fig. 4B), while the maximum ALD increased with climatic year and more intensely so for RCP 8.5 than for RCP 2.6 (Fig. 4C). Under RCP 8.5, ALD reached its maximum, meaning that surface permafrost disappeared under late 21st century climate. Surface runoff showed increasing trends under both RCP scenarios, while subsurface runoff only occurred under RCP 8.5 with the climatic conditions of 2075 and later (Fig. 4D).

### Sensitivity to the changing environment

Over the forecast 21st century climatic change, simulated larch growth was most sensitive to precipitation and temperature (results for all environmental variables are shown in Table 1). Higher precipitation increased annual NPP of larch and understory vegetation by 27.3% (+0.67 Mg C ha^−1^ year^−1^) and 64.8% (+0.44 Mg C ha^−1^ year^−1^), respectively, indicating that plant productivity is constrained by water availability during the growing season (Sugimoto et al. 2002; Dolman et al. 2004; Nikolaev et al. 2011a,b). Moreover, ALD decreased by 34.7% (−22.4 cm), which can be explained by the accompanying increases in aboveground litter (10.3%; +4.0 Mg C ha^−1^), soil wetness (24.2%), and LAI of both larch (15.0%; +0.16 m^2 ^m^−2^) and understory vegetation (58.3%; +0.28 m^2 ^m^−2^).

**Table 1:** Sensitivities of simulated ecosystem functions and forest structure to changes in climatic variables over the 21st century, as forecast under the IPCC's RCP scenario 8.5. Each row compares the averages of last 10 years of the 20th and 21st centuries. All values without units are presented as percentage (%). In the ALD column, “Max” indicates that ALD reached the deepest soil layer (2.0 m). “Full environmental items” include cumulative changes in all forcing environmental variables presented, as well as wind, humidity, and air pressure; as the effects of wind, humidity, and air pressure were not individually apparent, they are not presented separately. Forecast changes over the 21st century to each climatic variable are presented in parentheses at the tops of columns. The control condition is presented in parentheses at the left of rows. NPP is net primary production; LAI is leaf area index; ALD is active layer depth

	Precipitation (+175 mm year^−1^)	Air temperature (+10.4°C)	Air temperature × Precipitation	Atmospheric CO_2_ (+525 ppm)	Short and longwave radiations (−44 and +54 W m^−2^)	Full environmental items
Vegetation structures						
Annual NPP of larch tree (2.45 Mg C ha^−1^ year^−1^)	27.3	36.3	97.1	−10.6	−15.1	97.1
Annual NPP of understory vegetation (0.68 Mg C ha^−1^ year^−1^)	64.8	−61.8	−25.0	125.0	−33.8	−1.5
LAI of larch trees^a^ (1.01 m^2^ m^−2^)	15.8	55.4	87.1	−9.9	−5.9	87.1
LAI of understory vegetation^a^ (0.68 m^2^ m^−2^)	58.3	−37.5	−4.2	102.1	−25.0	22.9
Leafing days of larch trees (119.5 days)	1.3	44.8	44.9	−2.9	1.9	44.9
Aboveground litter (38.4 Mg C ha^−1^)	10.3	−30.6	−22.1	−20.5	−19.6	−19.7
Thermo‐hydrological properties						
ALD^a^ (64.4 cm)	−34.7	162.9	Max	−56.3	70.5	Max
Liquid state soil water content within 0–50 cm depth^a^ (0.199 m^3^ m^−3^)	24.2	−0.1	33.8	−9.0	−2.9	12.6
Surface runoff (14.8 mm year^−1^)	293.6	−27.4	73.8	−7.3	−30.6	55.0
Subsurface runoff (0 mm year^−1^)	NA	NA	39 mm year ^−1^	NA	NA	17 mm year ^−1^

a Mean values for June to August, which is the larch‐growing season in eastern Siberia under current climatic conditions.

Higher air temperature also increased the annual NPP of larch by 36.3% (+0.89 Mg C ha^−1^ year^−1^). Leaf flushing days extended by 44.8% (+53.5 days), which accounts for the greater productivity as changes in soil wetness (June–August) are negligible. In addition, the extended flushing of larch trees could also explain the decreased NPP of understory vegetation (−61.8%; −0.42 Mg C ha^−1^ year ^−1^). Despite the negligible changes in soil wetness, ALD increased by 162.9% (+105 cm), which is in accordance with higher air temperatures and less aboveground litter (−30.6%; −11.8 Mg C ha^−1^).

When precipitation and air temperature were changed together, the annual NPP of larch increased by 97.1% (+2.38 Mg C ha^−1^ year ^−1^). This effect is about 34% greater than for the simpler additive effect of precipitation (27.3%) and air temperature (36.3%), indicating that the interaction of an extended growing period with wetter soil conditions further increases larch productivity. Under combined precipitation and temperature changes, the surface‐active layer disappeared; subsurface runoff still occurred (39 mm year ^−1^), but soil wetness during the larch‐growing season (June to August) increased by 33.8%.

Elevated atmospheric CO_2_, which amplifies photosynthesis in the model via both a fertilization effect and improved water‐use efficiency, decreased the annual NPP of larch by 10.6% (−0.26 Mg C ha^−1^ year ^−1^) while increasing that of understory vegetation by 125% (+0.85 Mg C ha^−1^ year ^−1^). A shallow ALD may be responsible for these opposite reactions, as it would have provided more water to understory vegetation with an assumed root depth of 10 cm compared to larch trees with an assumed root depth of 50 cm. Indeed, soil wetness up to 50 cm depth decreased by 9.0% from June to August. This shallow ALD was likely caused by more aboveground litter (20.5%; +7.0 Mg C ha^−1^) and higher LAI (26.0%; +0.39 m^2^ m^−2^, average June–August).

Radiation changes reduced the annual NPP of larch and understory vegetation by 15.1% (–0.37 Mg C ha^−1^) and 33.8% (–0.23 Mg C ha^−1^), respectively, which can be explained by weaker shortwave radiation in the forecast climate. Finally, it is notable that changing all environmental factors simultaneously produced a similar result to changing only air temperature and precipitation, indicating that these two are the dominant factors regulating this ecosystem.

### Simulations for the entire larch‐dominated area

**Figure 5:**
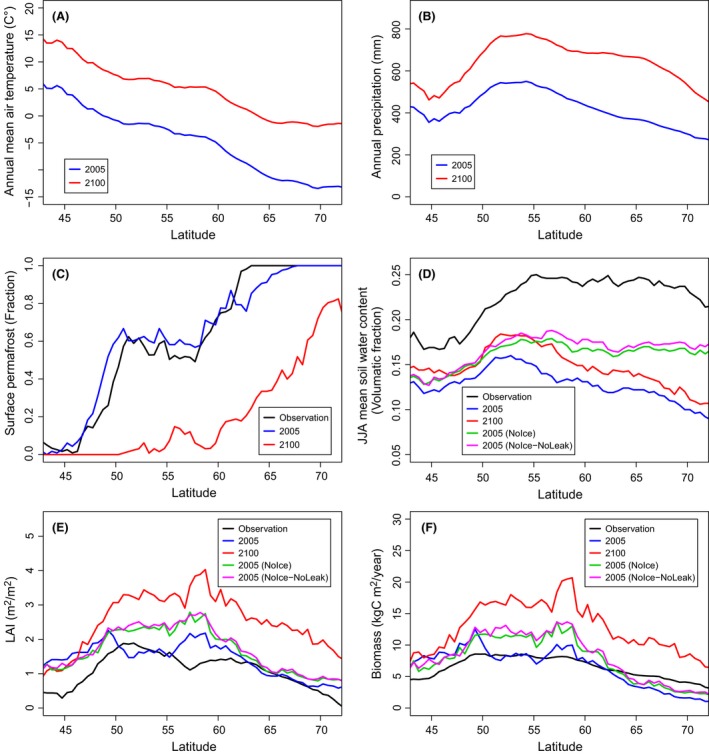
Latitudinal gradients of (A) annual mean of air temperatures, (B) annual precipitation, (C) fraction of surface permafrost, (D) mean soil moisture content from June to August, (E) tree LAI in July, and (F) biomass. Values are calculated for terrestrial grid cells within the rectangular area shown in Figure 1. Air temperature and precipitation are taken from the climatic data used for the simulations in this study. Simulations of years 2006–2100 used the MIROC‐based projection under RCP 8.5. For biomass, surface permafrost, and soil moisture, simulation outputs are compared with observation‐based estimates (sources in Table 2). Simulation results are shown as 10‐year averages by the years of 2005 and 2100 for standard simulations and 10‐year averages by the year of 2005 for the NoIce run.

The MIROC‐ESM climate projection under RCP 8.5 forecasts increasing trends in annual air temperature and precipitation for the entire larch‐dominated area during the 21st century (latitudinal averages predicted to increase by c. 8–12°C and c. 80–300 mm year^−1^, respectively; Fig. 5A and B). This climatic change trajectory is strongly supported by the Coupled Model Intercomparison Project Phase 5 (CMIP5) (IPCC, 2013): the multimodel mean of the CMIP5 shows both annual air temperature and precipitation across Siberian increasing under RCP 2.6, 4.0, 6.0, and 8.5.

**Table 2:** Data sets used for model validation in Figure 5

Data Set	Description	References	Note and URL
Surface permafrost	Circum‐Arctic Map of Permafrost and Ground‐Ice Conditions, Version 2	Brown et al. (2002)	Continuous, discontinuous, and sporadic permafrost are simply combined for the analysis. http://nsidc.org/data/ggd318.html
Surface soil moisture	ESA CCI Surface Soil Moisture Combined Product Version 2.2	Liu et al. (2012), Liu et al. (2011), Wagner et al. (2012)	Data during 1996–2005 are averaged www.esa-soilmoisture-cci.org
Leaf area index		Kobayashi et al. (2010)	Summer maximum (July) tree LAI averaged between 1998 and 2013. The tree LAI was derived by the SPOT‐VEGETATION S10 satellite data sets http://flies.sakura.ne.jp/WP/satellite-product/
Biomass	Global spatial data set for total (aboveground and belowground) biomass	Kindermann et al. (2008)	www.iiasa.ac.at/Research/FOR/biomass.html

Our model reconstructed well the latitudinal gradient of the area of surface permafrost, and forecast a retreat under RCP 8.5 warming (Fig. 5C). By the end of the 21st century, nearly no surface permafrost is predicted to remain in this region south of 60°N. For soil moisture (Fig. 5D), simulation outputs (soil moisture within larch root depth, 0–50 cm) cannot be directly compared with the satellite‐based estimates as the satellite gathers data from an area of hundreds of km^2^ using microwave sensors, which only detect available water within a thin surface soil layer (0.5–5 cm). Nonetheless, this satellite data displays a clear decreasing gradient in soil wetness southward from around 55°N, and our model also reconstructed this gradient. On the other hand, the model also showed a decreasing northward gradient of soil moisture starting from around 55°N, which is not visible in satellite data. However, this northward gradient was not present in the NoIce and the NoIce‐NoLeak runs, indicating that this pattern is primarily caused by inhibition of surface water infiltration by frozen soil layers. In the model, excess water for infiltration instantaneously disappears as surface runoff, but in reality, it would stay in the proximate area, and this mismatch explains the difference in outcomes.

Simulated soil moisture increased over the 21st century for the entire larch‐dominated area, despite finding no obvious trend in simulations at Spasskaya‐pad. This accords with the trend of increasing precipitation, besides which the reduction of surface runoff under warming would also bring about a similar pattern. Disappearance of the surface permafrost also causes bottom flow of soil water, but the effect that this would have on soil moisture and plant productivity would be minor as there were no major differences between the NoIce and NoIce‐NoLeak runs.

The model reconstructed the pattern of larch LAI and biomass decreasing toward the northern and southern borders of the larch‐dominated area (Fig. 5E and F). However, the southward pattern is stronger in the observation‐based data, such that the model overestimated larch LAI and biomass at the southern edge of the larch‐dominated zone. In the NoIce and the NoIce‐NoLeak runs, both LAI and biomass increased in the latitudinal range of 50–63°N, indicating that frozen soil layers are the primary limit on plant productivity in this region. By the end of the 21st century, both LAI and biomass increased throughout the entire larch‐dominated zone.

## Discussion

### Dependence of Siberian larch forest on near‐surface permafrost

We developed an integrated model of the response of larch forest to projected climatic changes, which was adapted and applied to larch forest at Spasskaya‐pad in Siberia. The model shows that annual larch NPP under RCP scenarios 2.6 and 8.5 is, respectively, about two and three times higher under climatic conditions of the end of 21st century compared with the 20th century. Sensitivity tests show that air temperature and precipitation are the primary factors contributing driving this increasing trend in larch NPP, although many processes those are not included in the model may constrain such a large productivity response (e.g., nitrogen limitation, waterlogged soil, heat stress, and vegetation change). The higher air temperatures forecast under RCP 8.5 increases larch annual NPP by 36.3%, which is partially attributable to the 44.8% extension the larch leafing period. Under these conditions, surface permafrost is maintained and there are no notable changes in soil water content, although ALD increases by 162.4%. Our model predicts that surface permafrost will only decay when air temperature and precipitation increase simultaneously, as indeed forecast by RCP 8.5. In this case, subsurface runoff occurs but the soil water available for plants increases at the same time, resulting in greater annual larch NPP.

Simulations across the entire larch‐dominating area also support a relatively small effect of surface permafrost disappearing on soil desiccation: both the NoIce and the NoIce‐NoLeak runs produce very similar latitudinal gradients of soil wetness, LAI, and tree biomass. Rather, the primary limit on plant productivity in the central latitudinal range of this region is the frozen soil layers, with both the NoIce and the NoIce‐NoLeak runs showing increases in soil wetness, LAI, and biomass at 50–63°N.

Our simulation is qualitatively consistent with the earlier simulation by Beer et al. (2007), which predicted an increase in larch biomass without considering freeze–thaw processes. In contrast, a previous simulation by Zhang et al. (2011) resulted in excess drainage, soil desiccation, and decay of larch forests. A frozen soil layer affects soil water content by inhibiting both drainage of soil water and infiltration of surface water. The simulation by Zhang et al. (2011) is more sensitive to the former process than ours was, whereas the simulation by Beer et al. (2007) does not consider this process whatsoever. The validity of our results is supported by our reasonable reproduction of observed latitudinal gradients in thermos‐hydrological properties. Furthermore, our results match an observed positive relationship between interannual variation in ALD and unfrozen near‐surface soil water content in the Yakutsk area (Iijima et al. 2010). An alternative explanation for this pattern has, however, been proposed, with Iijima et al. (2010) suggesting that more unfrozen soil water requires more time to freeze in early winter and thus maintains a higher soil temperature throughout winter, resulting in a deeper active layer in the next growing season.

### Generality of the simulations

Although the model was calibrated and validated for the Sppaskaya‐pad site, it reconstructed latitudinal gradients of permafrost existence, soil moisture, tree LAI, and biomass reasonably well across the entire larch‐dominated area in eastern Siberia, where increasing annual mean air temperature and annual precipitation are forecast. Projected regional changes in hydrology and plant productivity are also consistent with the simulation at Sppaskaya‐pad, although the areawide increase in soil wetness was not projected for Sppaskaya‐pad. Our site‐specific outcome can therefore be regarded as a typical example of a general phenomenon for most of the eastern Siberian larch‐dominated region.

Despite its good performance on hydrological and soil properties, the model overestimated larch LAI and biomass at the southern margin of the larch‐dominated zone. This border between forest and steppe exists primarily because of drought during the growing season (Dulamsuren et al. 2009, 2011); hence, the inconsistency between model outcomes and observed values indicates that the model overestimates soil wetness in this area. Consistent with this possibility, simulated interannual variation of mean summer soil water content at Sppaskaya‐pad was smaller than the observed variation. Refining this process in the model might dampen the projected higher plant productivity under a warming climate.

In contrast, our model may have underestimated the increase in productivity by not considering the ecological impact of decaying permafrost. In the northern part of the Siberian larch forest, thawing surface permafrost would in itself boost aboveground biomass: aboveground biomass is directly associated with the biomass of living roots, which is constrained by ALD. Indeed, the tree canopy is always thinner in northern open forests regardless of tree density, indicating that ALD is the predominant constraint on the potential maximum aboveground biomass in these areas (Sofronov et al. 2000). Incorporating this process into the model might thus accelerate the projected increase in plant productivity as the climate warms.

### Limitations of the model

Because our model overestimated changes in ALD after clear‐cutting and underestimated the range of interannual variation in soil wetness at Sppaskaya‐pad, there is still scope for improving its ability to reproduce thermo‐hydrology. For longer‐term predictions, we would also have to consider the possibility of thermokarst lakes growing as a result of ground ice melting and concomitant surface subsidence. This occurs due to the wide distribution of extremely ice‐rich permafrost with large ice wedges across the lowland of northeastern Siberia (Schirrmeister et al. 2002); the Lena River basin, where Sppaskaya‐pad is located, is underlain by ice‐rich sediments (average volumetric ice content of the sediment is 50%) with a massive ice wedge (Fedorov et al. 2014). A long‐term observational study on this region from 1992 to 2008 showed that melting ground ice, accelerated by current warming trend, caused thermokarst lakes to develop by collecting lateral water flow from the surrounding terrain (Fedorov et al. 2014). Because surface water bodies stimulate melting of ground ice and thus bring about surface subsidence, thermokarst lakes grow further as ground ice continues to melt. This is supported by remote sensing that has shown an increasing trend in the number and total area of surface water bodies in the continuous permafrost zone across Siberia (Smith et al. 2005; Grippa et al. 2007).

The large water quantities that collect in thermokarst lakes can have further impacts on larch forest by oversaturating the near‐surface soil. This can cause forest loss in the surrounding areas as excessively wet soil can cause hypoxia in larch roots and hence extremely slow gas exchange (Schulze et al. 2005). Indeed, perennially waterlogged conditions in Yakutsk from the winter of 2004, caused by increased precipitation, resulted in foliar suppression and browning on mature larches by 2007 (Iijima et al. 2014). Our model does not consider these negative effects of waterlogging on larch growth, and hence, our forecast increases in larch productivity under warming may overestimate averages values on a large geographical scale.

Another process that we did not consider and may limit the model is metabolic stress under high temperatures. Previous versions of the SEIB‐DGVM assumed the maximum growing degree days above 5°C (*GDD5*) required for establishment of *Larix gmelinii* is 1500 (Nikolaeva et al. 2004). We did not use this cutoff because this value of *GDD5* causes larch recruitment to stop abruptly in simulations at Sppaskaya‐pad under any RCP scenarios. Similarly, Shuman et al. (2011) showed that a *GDD5* of 1500 causes an abrupt collapse of larch forest in southern Siberia when annual average temperatures increase by 4°C. This *GDD5* value was estimated using the realized niche of plant species, which also depends on competition among tree species and thus does not imply fixed physiological constraints. In fact, the majority of tree species across arctic, temperate, and moist tropical habitat types generally show heat damage at 44–52°C (Loehle 1998). Indeed, transplanted *L. gmelinii* trees survived for over a hundred years in Sapporo, Japan (43°40′N, 141°21′E) (Hokkaido University Botanic Garden 2003), where annual mean temperature is 9.3°C and *GDD5* is 2469 (2001–2010 average, based on climate records from the Japan Meteorological Agency), which strongly suggests that such abrupt collapse due to heat stress is not a plausible scenario. Nonetheless, this does not rule out heat stress as a constraint on the growth of *L. gmelinii*, which may explain the model's overestimation of simulated larch biomass and LAI near the southern margin of the larch‐dominated area.

An established constraint on larch growth is mineral availability. Siberian larch forest generally suffers from severe nitrogen deficiency (Schulze et al. 1995), which limits leaf carbon gain (Vygodskaya et al. 1997). Our model does not consider nitrogen limitation, implying that nitrogen constraint does not set an upper limit for biomass increase in a changing environment. This assumption may, partially at least, be rationalize, because decomposition of soil carbon (including thawed permafrost carbon) and resultant nutrient release could increase the amount of carbon stored as biomass (Shaver et al. 2000). However, quantitative impact of this nutrient supply is not certain. Also, such growth enhancement may be inhibited by spatial (e.g., depth in the soil profile) or temporal (e.g., winter vs. summer) variation in the relationship between decomposition of soil carbon and plant nutrient uptake (Schuur et al. 2008).

In terms of vegetation dynamics in the model, we did not consider the possibility of forest composition changing with climate. In the simulation used by Zhang et al. (2011), drier environments caused the dominant tree species to shift from larch to more drought‐tolerant pine and birch (Nikolov and Helmisaari 1992). Conversely, our model predicted a moister environment, suggesting that flood‐tolerant species such as willow may become more common. Whatever the shift in dominant species, plant migration requires time‐consuming processes such as seed dispersal, competition with resident species, and growth of the invading plant species (Sato and Ise 2012). In fact, based on vegetation shifts during the Holocene, migration rates of spruce species are estimated to be only 8–50 km per century (Van Minnen et al. 2000), approximately the cell size of the 0.5 degree grid used in our simulation. Therefore, our simplification that excludes these compositional changes would be reasonable for projecting major changes in the Siberian larch forest over the next 100 years.

A final limitation of our model is our assumption that no fire during occurred the Sppaskaya‐pad simulation periods. In boreal ecosystems, predicted increases in air temperature may lengthen the fire season and increase the probability and frequency of wildfires (Randerson et al. 2006). Our simulation may thus overestimate biomass and productivity of larch forests, as wildfire is the most influential disturbance mechanism in boreal regions (Kasischke et al. 2005), determining whether arctic land functions as a sink or source for atmospheric CO_2_ (Mcguire et al. 2010).

### Implications of this study

Changes in boreal ecosystems can significantly affect the earth's albedo (Foley et al. 2003) and directly modify the global carbon balance because of their huge area and vast carbon pools stored as biomass and in the soil (Malhi et al. 1999; Tarnocai et al. 2009). Our findings are valuable as they reduce uncertainties regarding the future of these globally important larch forests and near‐surface permafrost in eastern Siberia, and at the same time highlight the need to consider complex biological mechanisms to accurately forecast local and global responses to climate change.

## Conflict of Interest

None declared.

## Supporting information


**Appendix S1** Details for model structures, forcing data, model calibration, and model validations.
**Table S1.** Fixed parameters used in the model.
**Table S2.** Abstract of the climate data for forcing the model.
**Figure S1.** Abstract of climate data used to force the model for the simulations after the 21st century at the Sppasksya‐pad.
**Figure S2.** A calibration result of the comparison between simulated and observed development of ALD_max_ (seasonal maximum of the active layer thickness) after stand‐replacing fire.
**Figure S3.** A calibration result of the comparison of simulated and observed post‐fire succession of carbon storage in the soil organic layer.
**Figure S4.** Simulated vegetation dynamics after a stand‐replacing fire at the Spasskaya‐pad.
**Figure S5.** Comparisons of simulated and observation‐based seasonal changes in ecosystem functions.
