# Supplementary material for: Endurance of larch forest ecosystems in eastern Siberia under warming trends
Source: Ecol Evol. 2016 Jul 22;6(16):5690–704. doi: 10.1002/ece3.2285 (PMC4983584; doi:10.1002/ece3.2285)
Supplement: Supplementary file 1 — Appendix S1 Details for model structures, forcing data, model calibration, and model validations. Table S1. Fixed parameters used in the model. Table S2. Abstract of the climate data for forcing the model. Figure S1. Abstract of climate data used to force the model for the simulations after the 21st century at the Sppasksya‐pad. Figure S2. A calibration result of the comparison between simulated and observed development of ALDmax (seasonal maximum of the active layer thickness) after stand‐replacing fire. Figure S3. A calibration result of the comparison of simulated and observed post‐fire succession of carbon storage in the soil organic layer. Figure S4. Simulated vegetation dynamics after a stand‐replacing fire at the Spasskaya‐pad. Figure S5. Comparisons of simulated and observation‐based seasonal changes in ecosystem functions. [file ECE3-6-5690-s001.docx]

# Supporting Information

## Overview of the SEIB-DGVM

In the current study, we used the modifications developed by Sato *et al.* (2010). These adapted the original SEIB-DGVM (Sato *et al.*, 2007) to an eastern Siberian larch forest by incorporating empirical rules of allometry, allocation, and phenology developed for larch trees at the Spasskaya-pad tower site, Yakutsk, Russia. Because previous reports provided a detailed description of the model (Sato *et al.*, 2007), we provide only a brief description of the major processes and modifications made in the present study.

The simulation unit of the model was a 30 × 30-m section of the spatially explicit virtual forest where individual trees were established, competed, and died. Trees were established as saplings 1 cm in diameter. Individual trees were composed of three organs: the crown and trunk, which were cylindrical, and the fine roots, which were formless. The model determined photosynthetic rates of trees by simulating light conditions and spatial relationships with other trees. A forest-floor vegetation layer existed under the tree canopy. Plant population dynamics in the virtual forest were considered within conditions dictated by the carbon and water cycles. The computational time step was daily for all physical and physiological processes and yearly for all plant population dynamics, such as tree establishment, mortality, and wildfires.

Larch was the only woody species in the model, aside from C3 understory plant functional types, which implicitly included all small trees and forest floor coverage. The background mortality of each individual tree was assumed to be *M*_1_ / *M*_2_ ^–^*^v^*, where *v* is the annual NPP divided by the annual mean leaf area (m^2^ tree^–1^ year^–1^), and *M*_1_ and *M*_2_ are calibration parameters. For trees with negative annual NPP in the previous year, background mortality became 10 times larger. If the parameter *M_2_* is calibrated to be 1.0, the background mortality becomes independent on *v*, but tree mortality is still under control of carbon starvation by this assumption. Stand-replacing fire was the only disturbance scheme incorporated in the model, besides gap formation, which was caused by the death of large trees. We assumed that fire consumed all AGB of the understory, all tree leaf and trunk biomass, and the entire forest litter pool, except fine-root litter. However, we further assumed that fire transformed fine-root biomass into litter. Note that an observation study showed that stand-replacing fire did consume most of the surface soil organic layer in this ecosystem; satisfactory larch regeneration only occurred when the organic layer decreased to 2–5 cm thick as a result of wildfires, whereas the layer in mature larch forest is typically 10–25 cm thick (Sofronov & Volokitina, 2010). Table S1 lists the fixed parameters used in model equations.

Litters from different origin would have different thermal conductivities, but we simply treated litter as a group in this parameterization, as distinguishing litter types would make the model structure too much complicated by considering differences of bulk density, moisture holding capacity, and decomposition rate among litter type.

The SEIB-DGVM simply treats floor vegetation with a generic C3 grass plant functional type. In reality, the forest floor of Sppaskaya-pad was dominated by dense cowberry in 1997, but shrubs and moisture-tolerance grasses become dominated during greater precipitation years from 2005 to 2008 (Ohta *et al.*, 2014). While, the ground surface of Siberian open forests is also usually dominated by feather-mosses and lichens (Sofronov *et al.*, 2000). Such temporal and spatial heterogeneity of the understory vegetation can modify the insulating efficiency for permafrost thaw, and hence can influence the model projections.

To control both photosynthetic rate and leaf phenology as functions of soil moisture content, the physiological status of water availability (*statwater*, 0.0–1.0) was defined as follows:

*statwater* = (*pool*_w_ – *W*_fi_) / (*W*_fi_ – *W*_wilt_), [eq. S1]

where *pool*_w_ represents the fraction of volumetric soil water content within the rooting depth (50 cm for larch trees and 10 cm for understory plants), *W*_fi_ represents soil moisture at field capacity, and *W*_wilt_ represents soil moisture at wilting point. The non-water-stressed photosynthetic rate of each plant functional type was multiplied by

2 × *statwater* – *statwater*^2^. [eq. S2]

This function reconstructed an observed phenomenon in which the ratio of actual evaporation to potential evapotranspiration has high sensitivity to soil water content near the wilting point, whereas sensitivity is reduced near field capacity (Dunne & Leopold, 1978). Using the last update of the SEIB-DGVM, we implemented this function and successfully reconstructed the vegetation gradient with aridity (i.e., desert, savannah, seasonal forest, and rain forest) on the African continent (Sato & Ise, 2012), where soil water content plays a vital role in vegetation production, similar in eastern Siberia.

To determine the leaf emergence date, we used a previously reported empirical function (Yamazaki *et al.*, 2007) that was developed for reproducing leaf phenology at Spasskaya-pad. This function assumes that leaf emergence begins when the temperature in the top soil layer exceeds 5°C, and the cumulative degree-days above 0°C exceeds 100. During the 14 days after leaf emergence, all stock resources are transformed into available resources at a constant rate. The leaf shedding process follows (Sato *et al.*, 2010), and when the 10-day average air temperature is less than 7°C, leaf defoliation begins, lasting for 14 days.

## Model Integration and Settings

Both the SEIB-DGVM and NOAH-LSM vertically divide the ground into 20 soil layers, each 10 cm in depth. Larch trees can absorb soil water from the unfrozen top five soil layers (0-50 cm) until their wilting points, and they transpire absorbed water. This assumption would be reasonable at our study site because only 1.5% of the root surface area is deeper than 50 cm (Ohta *et al.*, 2008). For the simulations under the present climatic conditions, the soil base temperature is assumed to be constant at −2.5 °C, based on previous observations (Vasiliev & Fedrov, 2003). For simulations under the projected climatic conditions, soil-base temperature was adjusted once a year so that its difference from the mean annual temperature at the deepest soil layer of the previous year was reduced by 10%. For albedo, 0.14 was applied for no snow coverage (determined for a larch forest without snow coverage; Budyko, 1956), and 0.37 was applied for full snow coverage (determined for maximum snow coverage in a deciduous needle-leaf forest; Barlage *et al.*, 2005); intermediate values were applied in proportion to the snow coverage simulated by NOAH-LSM.

In the integrated model, the amount of aboveground litter (sum of litter from tree leaves, AGB of the understory layer, and half of trunk biomass; denoted as *litter*_ag_ in Mg C ha^–1^) controlled the thermal conductivity of the top soil layer (*df*_1_, in W m^–1^ K^–1^) as follows:

*df*_1_ = 0.17 – 0.005 × *litter*_ag_, *df*_1_ ≥ 0.01, [eq. S3]

for which the coefficients are determined by calibration processes (see “Parameter Calibration” section for details). For vegetation type, which was used to determine canopy conductance and surface roughness in NOAH-LSM, “Needle-leaf deciduous trees (larch)” was selected, whereas “Bare soil” was selected after clear-cutting in the validation test (see below for details). Table S1 lists the fixed parameters for the integrated model.

## Time Step Conversion of Climate Data

The computation time steps of SEIB-DGVM and NOAH-LSM were 1 day and 30 min, respectively. Because the climate data shared by both models were daily, the integrated model converted the time step of daily climate data to 30-min data for inputting into NOAH-LSM (Fig. 2).

The manner of this conversion depended on the climatic parameters. For air temperature, the daily maximum and minimum air temperatures were first calculated from the mean air temperature and daily temperature range. We assumed that the daily maximum and minimum air temperatures occurred, respectively, at two-thirds of day length and at sunrise. These temperatures were connected with sine curves to obtain within-day variability.

With respect to the temporal distribution of daily precipitation, we assumed that half of the daily amount fell from 00:00–01:00 h, and the other half from 12:00–13:00 h. This assumption has successfully simulated hydrological and permafrost dynamics at the Spasskaya-pad flux tower site (Yamazaki *et al.*, 2007), even though the length of a rain event can affect the amount of intercepted rain fall (Zeng *et al.*, 2000).

We assumed that downward short-wave radiation began at sunrise, reached the daily maximum at midday, ceased at sunset, and changed in accord with sine-squared curves. Downward long-wave radiation was assumed to be constant within a day. For relative humidity, variability within each day was calculated from the specific humidity of the day and the estimated air temperature for each time step.

## Climate Data for Simulations in the Sppaskaya-pad site under Current Climate

For simulations in the Sppaskaya-pad site under current climate, 41 years (1966–2006) of observation-based climate data were repeatedly inputted, and atmospheric CO_2_ concentrations were fixed at 368 ppm, which was the value observed in 2000. These climate data were generated from meteorological observations at Yakutsk city (62.08°N, 129.75°E, 101 m above sea level) by Baseline Meteorological Data in Siberia Version 5 (BMDS5) (Yabuki *et al.*, 2011). This data set included daily measurements of meteorological variables for 59 years (1950–2008). However, we only used the selected 41 years because the first 16 years (1950–1965) lacked sunshine duration data required for estimating downward radiations, and the last two years (2007–2008) were characterized by abnormally wet conditions in the central Lena River basin of the Yakutsk area (Iijima *et al.*, 2010).

Missing values were filled with the value of the same day in the previous year. Data from leap-year days were deleted. Because the meteorological observation station in Yakutsk city is about 20 km away from the Spasskaya-pad flux tower site, we converted the BMDS5 data with regression equations describing the relationship for each meteorological element between the meteorological observation station and tower site (Yamazaki *et al.*, 2007). These regression equations were calculated on a daily basis for 1998–2000, when tower data were available.

Because the BMDS5 data do not contain radiation, it was estimated by empirical methods. The downward short-wave radiation was estimated from the duration of sunshine, and the downward long-wave radiation was calculated from the duration of sunshine, air temperature, and vapor pressure (Kondo *et al.*, 1991). Also, specific humidity was estimated from the minimum relative air humidity and maximum daily temperature, and was assumed to not change within a day.

## Parameter Calibration

To calibrate the model, the following three steps were repeated until calibration parameters become stable. Essentially, this calibration process only modified post-fire development of thermal conductivity in the top soil layer, and forest structure in terms of tree density and AGB.

### Step 1: Post-fire development of ALD_max_

Post-fire development of *ALD*_max_ (seasonal maximum of the active layer thickness, in m) was reconstructed by calibrating the relationship between *df*_1_ (thermal conductivity of the top soil layer) and *litter*_ag_ (aboveground litter density). For this, simulation of bare ground with the full 41-year meteorological data set was repeated four times (total 164 years). During these simulations, fire was not allowed to occur.

Equation S3 resulted from repeating steps 1–3 several times. Figure S2 compares the observed and simulated development of the *ALD*_max_ after stand-replacing fire. For the observed data, 1.27 m (the average *ALD*_max_ from 1998 to 2004 in a mature larch stand at the Spasskaya-pad; Ohta *et al.*, 2008) was multiplied by the relative variance of *ALD*_max_ during forest succession (estimated from measurements at 22 points around the Spasskaya-pad in forest patches of various ages; (Vasiliev & Fedrov, 2003)). The calibrated model roughly reconstructed the observed post-fire development of *ALD*_max_. In Zhang *et al.* (2011), simulated post-fire ALD development took less than 20 years to recover fully, which is too rapid, likely because they fixed heat conductance between the soil and atmosphere, and their model depended primarily on LAI development for ALD recovery.

### Step 2: Stand structure of mature forest

We reconstructed the observed stand structure of mature larch forests by calibrating the two mortality parameters of larch trees, *M*_1_ and *M*_2_. From the forest inventory data of Schulze *et al.* (1995), three typical larch forests at Yakutsk (stand age 125–131 years) were selected as calibration targets to reconstruct mean tree density and AGB through a 130-year simulation by repeatedly inputting the 41-year Spasskaya-pad meteorological data set. The resulting parameter values were 0.001 and 1.0 for *M*_1_ and *M*_2_, respectively. At the end of the simulation, these values gave a mean density of 1311 trees ha^–1^ and a mean AGB of 47.7 Mg C ha^–1^, both of which were within the range of their respective observed values (1175–1760 ha^–1^, average 1453 ha^–1^; 36.2–60.1 Mg C ha^–1^, average 46.5 Mg C ha^–1^).

### Step 3: Development of soil organic carbon on the forest floor after stand-replacing fire

We reconstructed the development of soil organic carbon on the forest floor after stand-replacing fire by arbitrarily changing the *FR*_ratio_ (biomass ratio of leaves to other organs) of understory C3 plant functional types. The calibration target was from Shibuya *et al.* (2004), who studied the amount of carbon storage in the organic layer (A0) in larch forests of various ages in Yakutsk. We repeated a 200-year simulation following a stand-replacing fire 10 times for a total simulation spanning 2000 years. Ten repeats of simulated post-fire developments were averaged to determine the general trend, which was compared with observational data. Figure S3 shows the result of this calibration, which used 0.12 for the *FR*_ratio_. Note that this calibration implicitly accounts for the effects of surface fire, which is estimated to be responsible about 80% of the area burned in Siberia (Conard & Ivanova, 1997), and indirect post-fire biogenic releases of greenhouse gases in the future, which is expected to be two to six times greater than direct emissions (Dixon & Krankina, 1993). Therefore, the calibrated *FR_ratio_* value does not have biological meaning.

## Model Validation 1 (Post-fire vegetation development)

We first tested whether the calibrated model adequately reconstructs post-fire vegetation development. A 200-year simulation following a stand-replacing fire was repeated 10 times successively for a total simulation spanning 2000 years. During the 200-year simulation, fire was not allowed to occur until the transition to the following repeat. Figure S4a shows changes in LAI on July 31 during the 200-year simulation. Understory vegetation quickly appeared after a fire and was gradually replaced by trees; understory LAI reached equilibrium at 0.7 after 100 years. Tree LAI increased over the initial 100 years and reached equilibrium at approximately 1.5, very close to the estimated value of 1.56 in this forest (Ohta *et al.*, 2008). The annual NPP of trees increased after fire and nearly stabilized at 2.5 Mg C ha^–1^ year^–1^ after approximately 100 years (Fig. S4b). Although the trajectory of tree NPP was roughly consistent with field observations, its equilibrium value was larger than the range of observation-based estimations for mature larch forests in Yakutsk (1.5, 2.0, and 2.4 Mg C ha^–1^ year^–1^), although such data are sparse and diverse. After 50 years, AGB increased (Fig. S4c), whereas tree density decreased (Fig. S4d), indicating that average tree size continually increased. The trajectory of tree AGB is consistent with field observations, but we should keep in mind that tree AGB at 130 years was calibrated so that it falls within the range of observations.

## Model Validation 2: seasonal changes in ecosystem functions in a mature forest

We tested whether the calibrated model adequately reconstructed seasonal changes in the ecosystem functions of a mature forest. Figures S5a and S5b compare simulated and observation-based estimates of seasonal changes in NPP and NEE. Estimated NPP was generated from ASCII data subsets of acceptable quality from Moderate Resolution Imaging Spectroradiometer (MODIS) Daily Photosynthesis Product MOD17A2 (Running *et al.*, 2000) for the Spasskaya-pad tower site from 2000 to 2006. The algorithm for this estimation is based on the concept of light-use efficiency proposed by (Monteith, 1972), which relates NPP to the amount of absorbed photosynthetically active radiation. Estimated NEE was delivered from flux observations at the Spasskaya-pad tower site, collected using the eddy covariance method (Ohta *et al.*, 2001). Both the simulated and estimated NPP decreased about halfway through the growing season (170–180 days into the year). Corresponding patterns were observed in both simulated and observed NEE. The same pattern was reported in observations (Dolman *et al.*, 2004) and simulations (Sato *et al.*, 2010) at this study site; these studies suggested that inhibited productivity halfway through the growing season was primarily due to soil water deficits. Accordingly, our simulated soil water surplus decreased nearly continuously (fig. S5d) throughout most of the growing season (until around day 240) (fig. S5c).

## Satellite-based larch overstory LAI for validating the model

For validating the simulation result at a large geographical area, we extracted the spatial and temporal variations in larch overstory LAI in Siberia from a satellite-based LAI produced by (Kobayashi *et al.*, 2010). This LAI data set was produced from SPOT-VEGETATION 10-day maximum composite surface reflectance data sets (S10). We used the 16 year’s SPOT-VEGTATION observation period from 1998 to 2013. The overstory LAI was estimated from the seasonal increases in normalized difference infrared index, NDII (Hardisky *et al.*, 1983). The relationship between an increase in NDII and larch overstory LAI was physically calibrated by the plant canopy radiative transfer simulation (Kobayashi & Iwabuchi, 2008). The regions in Siberia, where larch is a dominant land cover, were extracted from the Global Land Cover 2000 data sets (IES-Global-Environment-Monitoring-Unit, 2003). The original overstory LAI was estimated at the spatial resolution of SPOT-VEGETATION (1/112°) and each 10-day temporal interval. Three 10-day periods in July were averaged to obtain the summer maximum LAI. To compare the model results with satellite-based LAI data, we aggregated the satellite-based LAI over 0.5° by 0.5°. Each grid contains the average larch overstory LAI with respect to the larch dominant area.

## Table S1. Fixed parameters used in the model.

| Parameters | Symbols | Values | Units of measurement | Sources | |  |  |
| --- | --- | --- | --- | --- | --- | --- | --- |
| **Photosynthesis** |  |  |  |  | |  |  |
| Maximum photosynthetic rate | *P*_max_ | 26 ^*^ | μ mol CO_2_ m^–2^ s^–1^ | (Koike *et al.*, 2000) | |  |  |
| Control value of light-use efficiency | *LUE* | 0.05 | mol CO_2_ mol photon^–1^ | (Vygodskaya *et al.*, 1997) | |  |  |
| Optimal temperature | *T*_opt0_ | 21 ^†^ | °C | (Korzukhin *et al.*, 2004) | |  |  |
| Minimum temperature | *T*_min_ | −2 ^†^ | °C | (Korzukhin *et al.*, 2004) | |  |  |
| Maximum temperature | *T*_max_ | 38 ^†^ | °C | (Vygodskaya *et al.*, 1997) | |  |  |
| Michaelis-Menten coefficient of CO_2_ dependence | *KM* | 30 | μ mol mol^–1^ | (Koike *et al.*, 2000) | |  |  |
| **Growth respiration** |  |  |  |  | | | |
| Needle | *RG*_f_ | 1.56 | g DM g DM^–1^ | (Sato *et al.*, 2007) | | |  |
| Trunk | *RG*_s_ | 1.68 | g DM g DM^–1^ | (Sato *et al.*, 2007) | | |  |
| Fine root | *RG*_r_ | 1.34 | g DM g DM^–1^ | (Sato *et al.*, 2007) | | |  |
| **Nitrogen content** |  |  |  |  |  |  |  |
| Needle | *PN*_f_ | 0.016 | g N g DM^–1^ | (Schulze *et al.*, 1995) |  |  |  |
| Sapwood | *PN*_s_ | 0.0014 | g N g DM^–1^ | (Schulze *et al.*, 1995) |  |  |  |
| Fine root | *PN*_r_ | 0.0138 | g N g DM^–1^ | (Friend *et al.*, 1997) |  |  |  |
| **Other metabolic parameters** |  |  |  |  | |  |  |
| Specific leaf area | *SLA* | 0.006 ^*^ | m^2^ g DM^–1^ | (Shirota *et al.*, 2004) | |  |  |
| Ratio of needle to fine root biomass | *FR*_ratio_ | 0.35 ^‡^ | Ratio | (Kajimoto *et al.*, 1999) | |  |  |
| Sapwood diameter | - | 1.88 | cm | (Schulze *et al.*, 1995) | |  |  |
| Fine root turnover rate | *TO*_r_ | 0.20 ^‡, §^ | year^–1^ | (Kajimoto *et al.*, 1999, Matamala *et al.*, 2003) | |  |  |
| Root depth of larch trees | - | 50 | cm | (Sato *et al.*, 2007) | |  |  |
| Root depth of understory plants | - | 10 | cm | (Sato *et al.*, 2007) | |  |  |
| **Population dynamics** |  |  |  |  | |  |  |
| Establishment probability on vacant forest floor | *P*_establish_ | 0.013 | m^–2^ year^–1^ | Tuning parameter | |  |  |
| Minimum annual mean PAR for establishment under the tree canopy at midday | *PAR*_min_ | 350 | μ mol photons m^–2^ s^–1^ | Tuning parameter | |  |  |
| A parameter for mortality | *M*_1_ | 0.001 | (dimensionless) | Tuning parameter | |  |  |
| A parameter for mortality | *M*_2_ | 1.5 | (dimensionless) | Tuning parameter | |  |  |
| **Albedo** |  |  |  |  | |  |  |
| Larch forest (Snow free) | *Albedo_t_* | 0.14 | Fraction | (Budyko, 1956) | |  |  |
| Larch forest (Maximum snow coverage) | *Albedo_t_* | 0.37 | Fraction | (Barlage *et al.*, 2005) | |  |  |
| After clear-cutting (Snow free) | *Albedo_t_* | 0.19 ^¦^ | Fraction | (Hollinger *et al.*, 2009) | |  |  |
| After clear-cutting (Maximum snow coverage) | *Albedo_t_* | 0.69^††^ | Fraction | (Barlage *et al.*, 2005) | |  |  |
| **Soil properties** |  |  |  |  | |  |  |
| Soil moisture at saturation point at the “Medium-fine” degree of coarse soil | *W*_sat_ | 0.465 | m m^–1^ | (Zobler, 1986) | |  |  |
| Soil moisture at wilting point at the “Medium-fine” degree of coarse soil | *W*_wilt_ | 0.103 | m m^–1^ | (Zobler, 1986) | |  |  |

DM is dry matter; N is nitrogen.

^*^ Surface-area-based values from the literature were converted to the projection-area-based values.

^†^ Used for applying to the daily mean temperature, subtracting 2ºC from the original value, which was observed for instant air temperature. Here, 2ºC was the observed difference between daily mean temperature and mean day-time temperature during June, August, and September at the Spasskaya-pad tower site.

^‡^ The definition of fine roots in this study is roots less than 2 mm in diameter.

^§^ Observed composition of fine roots for each size class (Kajimoto *et al.*, 1999) is multiplied by the estimated turnover rates of fine root for each size class observed for *Pinus taeda* (Matamala *et al.*, 2003).

^¶^ A large value was assumed here because the original value (1500 in the Sato *et al.*, 2007) inhibits larch establishment at Yakutsk when forecasted climate during the 21^st^ century are applied.

^¦^ Observed value for grassland

^††^ Observed value for open shrub

## Table S2. Abstract of the climate data for forcing the model. Averages for the last 10 years of the observation-based values (1996–2005) are compared with their projected changes from 2091 to 2100 under two different representative concentration pathways (RCPs).

|  | Air Temperature | Precipitation | Specific Humidity | Shortwave radiation (midday) | Longwave radiation  (daily average) | Wind velocity | Atmospheric CO_2_ |
| --- | --- | --- | --- | --- | --- | --- | --- |
| Observed means during 1996~2005 | −10.5 °C | 267.2  mm year^–1^ | 0.0027  g g^–1^ | 401.8  W m^–2^ | 243.3  W m^–2^ | 2.5  m s^–1^ | 370  ppm |
| Projected changes until 2091~2100 (RCP 2.6) | 4.1 °C | 22.2 % | 38.8 % | −3.1 % | 6.9 % | −38.8 % | 14.5 % |
| Projected changes until 2091~2100 (RCP 8.5) | 10.4 °C | 65.6 % | 93.3 % | −10.9 % | 22.2 % | − 51.4 % | 141.8 % |

# Figure legends

## Figure S1: Abstract of climate data used to force the model for the simulations after the 21^st^ century at the Sppasksya-pad: (a) annual mean air temperature, (b) annual precipitation, (c) annual mean absolute humidity, and (d) annual mean downward short-wave (mid-day) and long-wave (daily average) radiations. These data were taken from the output of the MIROC-AGCM, which was forced using the RCP2.5 and RCP8.6 GHG emission scenarios.


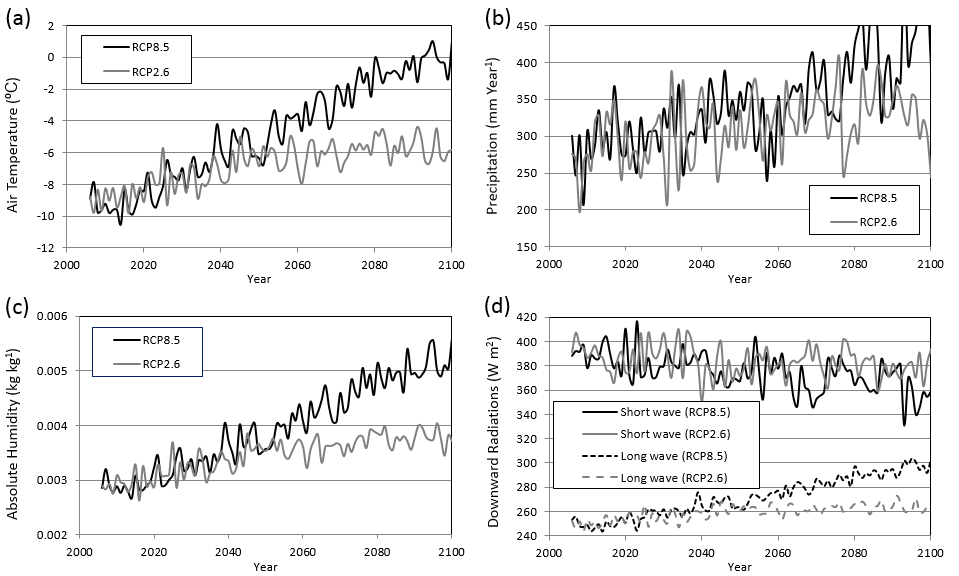


## Figure S2: A calibration result of the comparison between simulated and observed development of ALD_max_ (seasonal maximum of the active layer thickness) after stand-replacing fire. Observed values are from Ohta et al. (2008) and Vasiliev & Fedrov (2003) (see main text for details).


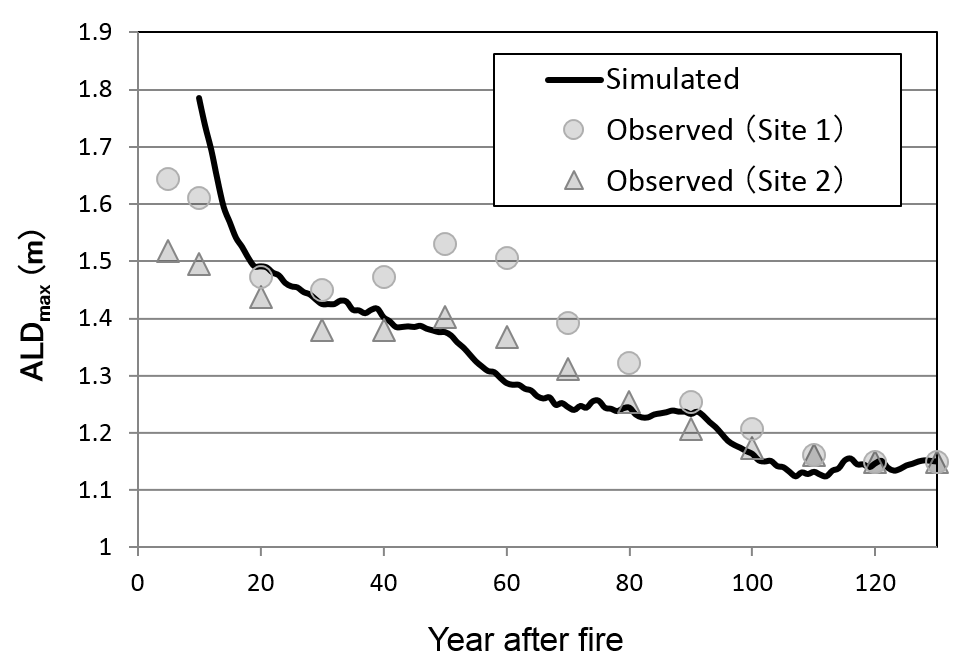


## Figure S3: A calibration result of the comparison of simulated and observed post-fire succession of carbon storage in the soil organic layer. Observed values are from Shibuya et al. (2004).


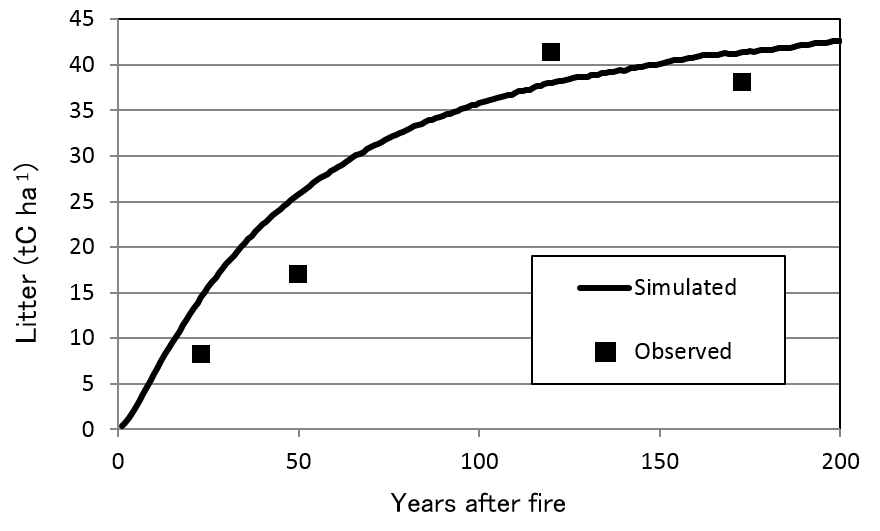


## Figure S4: Simulated vegetation dynamics after a stand-replacing fire at the Spasskaya-pad. Ten repetitions of independent simulations were averaged, and the 10-year running means are presented as follows: (a) LAI (leaf area index) of trees and understory vegetation on July 31; (b) NPP (net primary production) of larch trees; (c) tree aboveground biomass (AGB); and (d) tree density. Tree NPP and AGB were compared with field observations in Yakutsk. △Kajimoto et al. (2006); ▲Sawamoto et al. (2003); ●Schulze et al. (1995); □Shibuya et al. (2004). Sawamoto et al. (2003) provided only aboveground NPP, so these values were converted to total tree NPP using a time trend table of calculated annual net production rates of aboveground and belowground parts for larch stands in Siberia (Usoltsev et al., 2002).


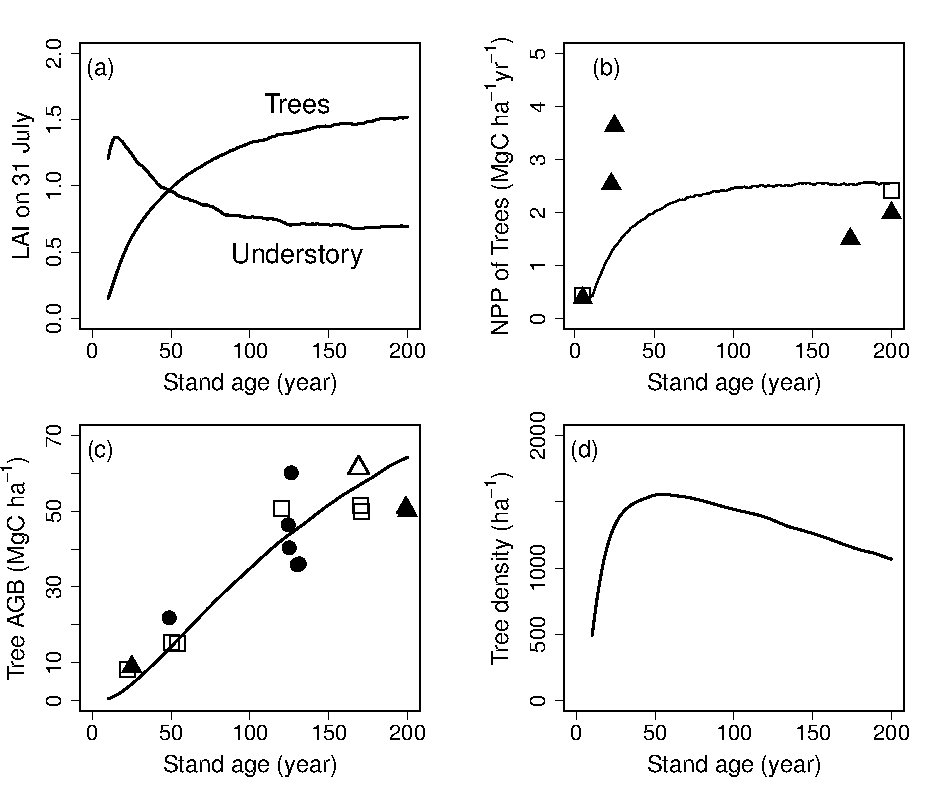


## Figure S5: Comparisons of simulated and observation-based seasonal changes in ecosystem functions. The 10-day running means are presented, and simulated results are the averages of 10 independent runs for a 160-year-old forest. (a) Simulated NPP compared with estimated NPP based on MODIS remote-sensing data (Running et al., 2000); (b) simulated NEE compared with estimated NEE based on carbon flux measurements at the Spasskaya-pad tower site (Ohta et al., 2001); (c) simulated larch GPP; (d) simulated water surplus calculated from the 100^th^ day of the year.


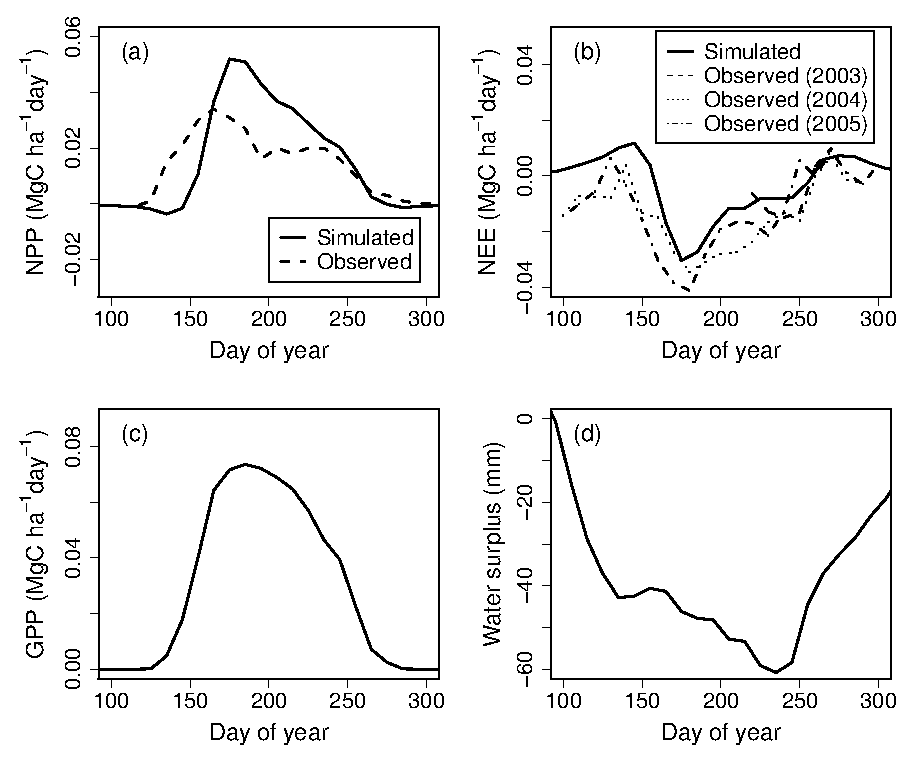


# References

Barlage M, Zeng XB, Wei HL, Mitchell KE (2005) A global 0.05 degrees maximum albedo dataset of snow-covered land based on MODIS observations. *Geophysical Research Letters,* **32**.

Budyko MI (1956) Heat Balance of the Earth's Surface. pp Page, Leningrad, Gidrometeoizdat.

Conard SG, Ivanova GA (1997) Wildfire in Russian boreal forests - Potential impacts of fire regime characteristics on emissions and global carbon balance estimates. *Environmental Pollution,* **98**, 305-313.

Dixon RK, Krankina ON (1993) Forest-Fires in Russia - Carbon-dioxide emissions to the Atmosphere. *Canadian Journal of Forest Research-Revue Canadienne De Recherche Forestiere,* **23**, 700-705.

Dolman AJ, Maximov TC, Moors EJ *et al.* (2004) Net ecosystem exchange of carbon dioxide and water of far eastern Siberian Larch (*Larix cajanderii*) on permafrost. *Biogeosciences,* **1**, 133-146.

Dunne T, Leopold LB (1978) Water use by Vegetation. In: *Water in environmental planning.*  pp Page. San Francisco, W. H. Freeman.

Friend AD, Stevens AK, Knox RG, Cannell MGR (1997) A process-based, terrestrial biosphere model of ecosystem dynamics (Hybrid v3.0). *Ecological Modelling,* **95**, 249-287.

Hardisky MA, Klemas V, Smart RM (1983) The Influence of Soil-Salinity, Growth Form, and Leaf Moisture on the Spectral Radiance of Spartina-Alterniflora Canopies. *Photogrammetric Engineering and Remote Sensing,* **49**, 77-83.

Hollinger DY, Ollinger SV, Richardson AD *et al.* (2009) Albedo estimates for land surface models and support for a new paradigm based on foliage nitrogen concentration. *Global Change Biology,* **16**, 696-710.

Ies-Global-Environment-Monitoring-Unit (2003) Global land cover 2000 database. (ed Cent. ECJR) pp Page, Ispra, Italy.

Iijima Y, Fedorov AN, Park H, Suzuki K, Yabuki H, Maximov TC, Ohata T (2010) Abrupt Increases in Soil Temperatures following Increased Precipitation in a Permafrost Region, Central Lena River Basin, Russia. *Permafrost and Periglacial Processes,* **21**, 30-41.

Kajimoto T, Matsuura Y, Osawa A *et al.* (2006) Size-mass allometry and biomass allocation of two larch species growing on the continuous permafrost region in Siberia. *Forest Ecology and Management,* **222**, 314-325.

Kajimoto T, Matsuura Y, Sofronov MA, Volokitina AV, Mori S, Osawa A, Abaimov AP (1999) Above- and belowground biomass and net primary productivity of a *Larix gmelinii* stand near Tura, central Siberia. *Tree Physiology,* **19**, 815-822.

Kobayashi H, Delbart N, Suzuki R, Kushida K (2010) A satellite-based method for monitoring seasonality in the overstory leaf area index of Siberian larch forest. *Journal of Geophysical Research-Biogeosciences,* **115**.

Kobayashi H, Iwabuchi H (2008) A coupled 1-D atmosphere and 3-D canopy radiative transfer model for canopy reflectance, light environment, and photosynthesis simulation in a heterogeneous landscape. *Remote Sensing of Environment,* **112**, 173-185.

Koike T, Yazaki K, Funada R *et al.* (2000) Photosynthetic characteristics of Dahurian larch, Scotch pine and white birch seedlings native to eastern Siberia raised under elevated CO_2_. *Eurasian Journal of forest research,* **1**, 31-37.

Kondo J, Nakamura T, Yamazaki T (1991) Estimation of the solar and downward atmospheric radiation. *Tenki,* **38**, 41-48.

Korzukhin MD, Vygodskaya NN, Milyukova IM, Tatarinov FA, Tsel'niker YL (2004) Application of a coupled photosynthesis-stomatal conductance model to analysis of carbon assimilation by spruce and larch trees in the forests of Russia. *Russian Journal of Plant Physiology,* **51**, 302-315.

Matamala R, Gonzalez-Meler MA, Jastrow JD, Norby RJ, Schlesinger WH (2003) Impacts of fine root turnover on forest NPP and soil C sequestration potential. *Science,* **302**, 1385-1387.

Monteith JL (1972) Solar radiation and productivity in tropical ecosystems. *The Journal of Applied Ecology,* **9**, 747-766.

Ohta T, Hiyama T, Tanaka H, Kuwada T, Maximov TC, Ohata T, Fukushima Y (2001) Seasonal variation in the energy and water exchanges above and below a larch forest in eastern Siberia. *Hydrological Processes,* **15**, 1459-1476.

Ohta T, Kotani A, Iijima Y *et al.* (2014) Effects of waterlogging on water and carbon dioxide fluxes and environmental variables in a Siberian larch forest, 1998-2011. *Agricultural and Forest Meteorology,* **188**, 64-75.

Ohta T, Maximov TC, Dolman AJ *et al.* (2008) Interannual variation of water balance and summer evapotranspiration in an eastern Siberian larch forest over a 7-year period (1998-2006). *Agricultural and Forest Meteorology,* **148**, 1941-1953.

Running SW, Thornton PE, Nemani AR (2000) Global terrestrial gross and net primary productivity from the earth observing system. In: *Methods in Ecosystem Science.* (eds Sala OE, Jackson RB, Mooney HA, Howarth RW) pp Page. New York, Springer-Verlag.

Sato H, Ise T (2012) Effect of plant dynamic processes on African vegetation responses to climate change: Analysis using the spatially explicit individual-based dynamic global vegetation model (SEIB-DGVM). *Journal of Geophysical Research-Biogeosciences,* **117**.

Sato H, Itoh A, Kohyama T (2007) SEIB-DGVM: A new dynamic global vegetation model using a spatially explicit individual-based approach. *Ecological Modelling,* **200**, 279-307.

Sato H, Kobayashi H, Delbart N (2010) Simulation study of the vegetation structure and function in eastern Siberian larch forests using the individual-based vegetation model SEIB-DGVM. *Forest Ecology and Management,* **259**, 301-311.

Sawamoto T, Hatano R, Shibuya M, Takahashi K, Isaev AP, Desyatkin RV, Maximov TC (2003) Changes in net ecosystem production associated with forest fire in Taiga ecosystems, near Yakutsk, Russia. *Soil Science and Plant Nutrition,* **49**, 493-501.

Schulze ED, Schulze W, Kelliher FM *et al.* (1995) Aboveground biomass and nitrogen nutrition in a chronosequence of pristine Dahurian Larix stands in eastern Siberia. *Canadian Journal of Forest Research-Revue Canadienne De Recherche Forestiere,* **25**, 943-960.

Shibuya M, Saito H, Sawamoto T *et al.* (2004) Time trend in aboveground biomass, net primary production, and carbon storage of natural *Larix gmelinii* stands in eastern Siberia. *Eurasian Journal of forest research,* **7**, 67-74.

Shirota T, Saito H, Takahashi K, Maximov TC, Maksimov A, Kononov AV (2004) Estimation of the productive structure of *Larix cajanderi* forest in Spasskaya Pad. *Proceedings of the International Semi-Open Workshop "C/H2O/Energy Balance and Climate over Boreal Regions with Special Emphasis on Eastern Eurasia"*, 39-42.

Sofronov MA, Volokitina AV (2010) Wildfire Ecology in Continuous Permafrost Zone. In: *Permafrost Ecosystems: Siberian Larch Forests.* (eds Osawa A, Zyryanova OA, Matsuura Y, Kajimoto T, Wein RW) pp Page. Berlin, Springer-Verlag GmbH.

Sofronov MA, Volokitina AV, Kajimoto T, Matsuura Y, Uemura S (2000) Zonal Peculiarities of Forest Vegetation Controlled by Fires in Northern Siberia. *Eurasian Journal of forest research,* **1**, 51-57.

Usoltsev VA, Koltunova AI, Kajimoto T, Osawa A, Koike T (2002) Geographical gradients of annual biomass production from larch forests in northern Eurasia. *Eurasian Journal of forest research,* **5**, 55-62.

Vasiliev IS, Fedrov AN (2003) Thaw depth variation with plant succession after anthropogenic disturbances, central Yakutia. In: *8th International Conference on Permafrost.* (eds Philips M, Springman SM, Arenson LU) pp Page, Zurich, Switzerland, Swets & Zeitlinger, Lisse.

Vygodskaya NN, Milyukova I, Varlagin A *et al.* (1997) Leaf conductance and CO2 assimilation of Larix gmelinii growing in an eastern Siberian boreal forest. *Tree Physiology,* **17**, 607-615.

Yabuki H, Park H, Kawamoto H, Suzuki R, Razuvaev VN, Bulygina ON, Ohta T (2011) Baseline Meteorological Data in Siberia (BMDS) Version 5.0, RIGC, JAMSTEC, Yokosuka, Japan, distributed by CrDAP, Digital media. pp Page.

Yamazaki T, Ohta T, Suzuki R, Ohata T (2007) Flux variation in a Siberian taiga forest near Yakutsk estimated by a one-dimensional model with routine data, 1986-2000. *Hydrological Processes,* **21**, 2009-2015.

Zeng N, Shuttleworth JW, Gash JHC (2000) Influence of temporal variability of rainfall on interception loss. Part I. Point analysis. *Journal of Hydrology,* **228**, 228-241.

Zhang N, Yasunari T, Ohta T (2011) Dynamics of the larch taiga-permafrost coupled system in Siberia under climate change. *Environmental Research Letters,* **6**.

Zobler L (1986) *A world soil file for global climate modeling*, National Aeronautics and Space Administration, Goddard Space Flight Center, Institute for Space Studies.
